# Health for all: a pathway to economic growth in the Association of South-East Asian Nations

**DOI:** 10.2471/BLT.14.138230

**Published:** 2014-11-01

**Authors:** Pat Oungpasuk

**Affiliations:** a Health Systems Research Institute, National Health Building (4th Floor), 88/39 Tiwanon 14 Road, Muang, Nonthaburi 11000, Thailand.

If the Association of South-East Asian Nations (ASEAN) were one country, its gross domestic product would be the seventh largest in the world.^1^ The launch of the ASEAN Economic Community in 2015 will reduce tariffs, increase the flow of goods, services, labour and capital between the Association’s member states, and lead to greater economic integration and improved economic outcomes.^2^ However, such gains will not be realized fully unless ASEAN’s labour force is first strengthened.

In any country, a strong and able labour force may allow national prosperity by increasing overall productivity and helping the government and the country to thrive via positive economic outcomes. The country’s policy-makers must not only create economic opportunities but also ensure that the national labour force is capable of seizing such opportunities.

In 2011, the European Higher Education Area was launched. The main aim was to increase integration between countries – by establishing a universal set of higher-education standards, decreasing differences and inequities in the education and the skill set of national workforces and facilitating greater labour movement.^3^ While observing the progress being made in Europe, Professor Dr Somkiat Wattanasirichaigoon – who is Director of the Health Systems Research Institute in central Thailand – has been championing the idea of using health as the cornerstone of a strategy to harmonize the member states of the future ASEAN Economic Community. For example, if universal health coverage could be established in each of the Community’s member states, the inequities within and between national populations and national workforces would probably be reduced. It is also likely that universal health coverage would strengthen ASEAN’s three main pillars – i.e. sociocultural integration, regional security and economic integration.

It seems sensible to improve the general well-being of a population – especially in low- and middle-income members of ASEAN – before progressing to the improvement of education and the development of useful skill sets. Unhealthy children are unable to reap the full benefits of education – because they have higher rates of absenteeism and poorer cognitive development than their healthy counterparts – and, in consequence, tend to grow into adults with relatively low earnings and productivity.^4^^,^^5^ The effective treatment of illnesses that cause high morbidity among children – e.g. malaria and hookworm – can substantially improve educational attainment, labour force participation and wealth.^6^

Health-related investments – whether targeted at children, adults or all age groups –contribute positively to economic growth.^5^^,^^6^ If, for example, these investments increase life expectancy, then they may also increase savings, spending on education and productivity. The treatment and eradication of hookworm in India led to increased earnings in the working population.^7^ In the United States of America, researchers found that, in the year 2003, illness led to a loss in productivity that had an estimated value of 260 billion United States dollars (US$).^8^

In several countries in Europe and in Thailand, the establishment of universal health coverage took 30–50 years.^9^ For the year 2000, it was estimated that the delivery of a very basic package of health services would cost about US$ 34 per person.^9^ When applied nationwide, such a cost might prove challenging to some of ASEAN’s member states, especially when the economic benefits may not be recognized for many years. However, such costs are not beyond the reach of any ASEAN member state. At the moment, political commitment – to create the structural reforms necessary to set the wheels in motion – appears to be as important as any financial commitment. The sooner universal health coverage can be implemented in member states, the sooner ASEAN can reap the benefits of increased competitiveness, increased domestic, regional and international security and better general health.

If ASEAN’s leaders want the ASEAN Economic Community to be sustainable and successful, it is critical that they start viewing health as human capital and universal health coverage as an effective pathway to economic outcomes that are both positive and sustainable. Although the cost of establishing universal health coverage may initially be high, such coverage will allow ASEAN’s member states to develop the strong base that they need to prosper both economically and developmentally.

## References

[1] World Economic Outlook Database [Internet]. Washington: International Monetary Fund; 2014. Available from: http://www.imf.org/external/pubs/ft/weo/2014/01/weodata/index.aspx [cited 2014 Jun 6].

[2] ASEAN Economic Community [Internet]. Jakarta: Association of Southeast Asian Nations Secretariat; 2014. Available from: http://www.asean.org/communities/asean-economic-communityhttp://www.imf.org/external/pubs/ft/weo/2014/01/weodata/index.aspx [cited 2014 Jun 6].

[3] Bologna Process – European Higher Education Area [Internet]. Yerevan: Bologna Follow-Up Group Secretariat; 2010. Available from: http://www.ehea.info/article-details.aspx?ArticleId=3 [cited 2014 Jun 28]

[4] Noronha K, de Figueiredo L, Andrade MV. Health and economic growth among the states of Brazil from 1991 to 2000. Rev Bras Estud Popul. 2010; 27(2):269–83 10.1590/S0102-30982010000200003

[5] Spence M, Lewis M, editors. Health and growth: Commission on Growth and Development. Washington: World Bank; 2009 10.1596/978-0-8213-7659-1

[6] Bloom DE, Canning D, Jamison DT. Health, wealth, and welfare. Finance Dev. 2004;41:10–5.

[7] Cutler D, Fung, W, Kremer, M, Singhal, M, Vogl, T. Mosquitoes: the long-term effects of malaria eradication in India [Working Paper 13539]. Cambridge: National Bureau of Economic Research; 2007. Available from: http://www.nber.org/papers/w13539.pdf [cited 2014 Jun 12]

[8] Davis K, Collins SR, Doty MM, Ho A, Holmgren AL. Health and productivity among US workers. Issue Brief (Commonw Fund). 2005(856):1–10. 16138438

[9] Carrin G, Mathauer I, Xu K, Evans DB. Universal coverage of health services: tailoring its implementation. Bull World Health Organ. 2008;86(11):857–63. 10.1126/science.1115717 19030691 PMC2649543

